# The fatty acid-rich fraction of *Eruca sativa* (rocket salad) leaf extract exerts antidiabetic effects in cultured skeletal muscle, adipocytes and liver cells

**DOI:** 10.1080/13880209.2017.1280687

**Published:** 2017-01-23

**Authors:** Mona H. Hetta, Asmaa I. Owis, Pierre S. Haddad, Hoda M. Eid

**Affiliations:** a Department of Pharmacognosy, Faculty of Pharmacy, Fayoum University, Fayoum, Egypt;; b Department of Pharmacognosy, Faculty of Pharmacy, Beni-Suef University, Beni-Suef, Egypt;; c Department of Pharmacology and Physiology, Natural Health Products and Metabolic Diseases Laboratory, Université de Montréal, Montréal, Quebec, Canada;; d Canadian Institutes of Health Research Team in Aboriginal Antidiabetic Medicines and Montreal Diabetes Research Center, Montréal, Quebec, Canada

**Keywords:** Brassicaceae, GC-MS, insulin resistance, type 2 diabetes, glucose uptake, adipogenesis, glucose-6-phosphatase

## Abstract

**Context:***Eruca sativa* Mill. (Brassicaceae), commonly known as rocket salad, is a popular leafy-green vegetable with many health benefits.

**Objective:** To evaluate the antidiabetic activities of this plant in major insulin-responsive tissues.

**Materials and methods:** Five *E. sativa* leaf extracts of varying polarity were prepared (aqueous extract, 70% and 95% ethanol extracts, the *n*-hexane-soluble fraction of the 95% ethanol extract (ES3) and the defatted 95% ethanol extract). *Eruca sativa* extracts were investigated through a variety of cell-based *in vitro* bioassays for antidiabetic activities in C2C12 skeletal muscle cells, H4IIE hepatocytes and 3T3-L1 adipocytes. Guided by the results of these bioassays, ES3 was fractionated into the saponifiable (SM) and the unspaonifiable (USM) fractions. Glucose uptake was measured using [^3^H]-deoxy-glucose, while the effects on hepatic glucose-6-phosphatase (G6Pase) and adipogenesis were assessed using Wako AutoKit Glucose and AdipoRed assays, respectively.

**Results:** ES3 and its SM fraction significantly stimulated glucose uptake with EC_50_ values of 8.0 and 5.8 μg/mL, respectively. Both extracts significantly inhibited G6Pase activity (IC_50_ values of 4.8 and 9.3 μg/mL, respectively). Moreover, ES3 and SM showed significant adipogenic activities with EC_50_ of 4.3 and 6.1 μg/mL, respectively. Fatty acid content of SM was identified by GC-MS. *trans*-Vaccenic and palmitoleic acids were the major unsaturated fatty acids, while palmitic and azelaic acids were the main saturated fatty acids.

**Discussion and conclusion:** These findings indicate that ES3 and its fatty acid-rich fraction exhibit antidiabetic activities in insulin-responsive cell lines and may hence prove useful for the treatment of type 2 diabetes.

## Introduction

The twenty-first century has witnessed a sharp increase in the number of people diagnosed with diabetes mellitus (DM). The International Diabetes Federation (IDF) estimated that 387 million people lived with DM in 2014. If no countermeasures are taken, this figure is expected to rise to 592 million in 2035 (IDF 2015). In the next 15 years, DM will be the seventh leading cause of death, mostly in low and middle-income countries (WHO: Diabetes Fact sheet 2015). The current prevalence of DM in the Middle East and North Africa (MENA) region is 9.7% (IDF 2015). Egypt has the highest prevalence of the disease, exceeding the international rate to reach 20% in urban areas (Herman et al. 1995).

Insulin resistance, defined as impaired insulin action in insulin-responsive tissues (mainly skeletal muscle, liver and adipose tissue), precedes the development of hyperglycaemia by many years or decades. It is caused primarily by ectopic fat accumulation in the skeletal muscle and liver (Yki-Jarvinen 2002). In the skeletal muscle, the primary site for postprandial glucose uptake, insulin resistance leads to impairment of insulin-stimulated glucose uptake and disposal. On the other hand, hepatic insulin resistance leads to increased hepatic production of glucose (gluconeogenesis) and, consequently, fasting hyperglycaemia in type 2 DM patients. The dephosphorylation of glucose-6-phosphate to generate glucose is the rate-limiting step of gluconeogenesis, which is catalyzed by glucose-6-phosphatase (G6Pase) (Rui 2014). Adipose tissue, once thought to be merely a storage organ, is now proved to be an active secretory organ that has an important role to play in controlling energy homeostasis and insulin sensitivity. Thiazolidinediones, a class of oral antidiabetic agents, improve insulin resistance mainly through promotion of adipogenesis and reduction of free fatty acid influx into skeletal muscle and liver (Chiarelli & Di Marzio 2008).

*Eruca sativa* Mill. (Brassicaceae), an edible plant of the mustard family, is commonly known as rocket salad, arugula, or taramira. The raw leaves are eaten fresh in salads or used as a spice. Its major chemical compounds are glucosinolates (GSL) and flavonoids (Michael et al. 2011). Glucoraphanin accounts for about 50% of total GSL in *E. sativa* leaves (Villatoro-Pulido et al. 2013). In addition, 4-methylthiobutyl GSL and 4-methylsulfinylbutyl GLS were also detected but at lower concentrations (Bennett et al. 2002). The total content of GSL was estimated to be 14.02-28.24 μM/g of dry weight (Villatoro-Pulido et al. 2013). The main flavonoids in *E. sativa* are kaempherol, isorhamnetin, rhamnocitrin, quercetin and their glycosides (Kim et al. 2014). Campesterol, β-sitosterol and brassicasterol are the most abundant sterols in the crude extract of the aerial parts and roots of *E. sativa*. In addition, triterpenes, mainly β-amyrin, were also detected but at lower concentrations (Khoobchandani et al. 2011). Essential oil of the leaves is rich in nitrogen and sulphur-containing compounds including isothiocyanate and 5-methylthiopentyl isothiocyanate (Jirovetz et al. 2002). The seed oil contains high amounts of erucic acid (46.64–54.79%), oleic (17.86–19.95%), palmitic (7.25–10.97%), linoleic (4.23–9.72%) and linolenic (1.98–3.01%) acids (Ugur et al. 2010).

In addition to its nutritional value, several health benefits are attributed to *E. sativa* such as antioxidant effects (Heimler et al. 2007), suppression of inflammatory cytokines (Kim et al. 2014), anticancer activity against melanoma cells (Khoobchandani et al. 2011) as well as antiplatelet, antithrombotic (Fuentes et al. 2014) and antiulcer activities against induced gastric lesions (Alqasoumi et al. 2009).

There is scarce information about the antidiabetic activity of the plant. One study reported that oral administration of the oil from the seeds resulted in the improvement of hyperglycaemia and lipid profile in rats (El-Missiry & El Gindy 2000). In addition, the inhibitory effect of water and ethanol extracts of fresh leaves on carbohydrate-hydrolyzing enzymes was reported (Hetta et al. 2014). Therefore, we carried out an *in vitro* screening of the different extracts of *E. sativa* fresh leaves for stimulation of glucose uptake, inhibition of G6Pase and adipogenic activities in order to evaluate their antidiabetic potential and to identify chemical components responsible for the antidiabetic activities.

## Materials and methods

### Plant material and extraction

The leaves of *E. sativa* were collected in September 2012 from Beni-Suef governorate, Egypt, and authenticated by Dr Ahmed Abdelhalim, botanist in Faculty of Science, Beni-Suef University, using traditional identification methods. A voucher specimen (no. BUPD 33) was deposited in the Department of Pharmacognosy, Faculty of Pharmacy, Beni-Suef University. The fresh leaves of *E. sativa* were cut into small pieces and extracted with organic solvents of different polarities in order to determine the potential biological activity of polar and non-polar components, including saponifiable and unsaponifiable ones. Firstly, 100 g of fresh leaves were cold-macerated for 24 h in ethanol 70% v/v (500 mL ×3) to give ES1 extract. A similar cold maceration method of extraction using 95% ethanol was adopted to prepare ES2 extract from 100 g of fresh leaves. ES3 extract was prepared by exhaustively defatting ES2 with *n*-hexane followed by evaporation of hexane. The remaining defatted residue was designated as ES4 extract. Finally, ES5 extract was prepared by the extraction of 100 g of the fresh leaves with distilled water (100 mL ×3). The organic solvents were distilled off under reduced pressure using a rotary evaporator (Büchi R-210, Switzerland). Water was removed by freeze-drying using ALPHA 1-2 LD plus freeze dryer (Fisher Bioblock Scientific, France). All the samples were kept in tightly closed containers at −20 °C until use. Yields of the different extracts are mentioned in Table 1. Based on the cytotoxicity and biological activity (described below), the active extract was subjected to further investigations.

**Table 1.** Percentage yield of the different extracts and fractions of *E. sativa* Mill.

Extract	% Yield
70% Ethanol (ES1)	9%
95% Ethanol (ES2)	6.5%
*n*-Hexane (ES3)	93% (of ES2)
Defatted 95% ethanol (ES4)	7% (of ES2)
Aqueous (ES5)	6%
Unsaponifiable matter (USM)	11.5% (of ES3)
Saponifiable matter (SM)	26.9% (of ES3)

### Cell culture

Murine C2C12 skeletal muscle myoblasts, H4IIE hepatoma cells and 3T3-L1 adipocytes were purchased from American Type Culture Collection (Rockville, MD) and were seeded in 12 well-plates in media containing 0.5% antibiotics (PS: Penicillin 100 U/mL, Streptomycin 100 μg/mL). C2C12 cells were grown in Dulbecco’s Modified Eagle’s Medium (DMEM) supplemented with 10% Foetal Bovine Serum (FBS), 10% Horse Serum (HS). To induce differentiation, myotubes were switched to 2% HS in DMEM for 7 days after reaching 80% confluence. H4IIE hepatocytes were grown in DMEM containing 10% FBS and experiments were performed when cells were fully confluent. 3T3-L1 cells were seeded in DMEM medium supplemented with 10% FBS (proliferation medium). To induce differentiation, two-day post-confluent cells were switched to differentiation medium (10% FBS, 500 μM 3-isobutyl-1-methylxanthine (IBMX), 500 nM insulin and 10 μM dexamethasone in DMEM) for 2 days. This medium was then changed every two day to fresh DMEM containing 10% FBS and 500 nM insulin and experiments were performed 8 days after the induction of differentiation. Cell culture media were purchased from Invitrogen Life Technologies (Burlington, Ont., Canada). All cells were incubated at 37 °C and 5% CO_2_. Plant extracts and fractions were dissolved in DMSO and diluted in 1:1000 for a final DMSO concentration of 0.1%. To screen for the various antidiabetic activities, cells were incubated with the maximum non-toxic concentrations of *E. sativa* extracts and fractions, as determined by the lactate dehydrogenase (LDH) assay (described below). For the determination of EC_50_ and IC_50_, cells were incubated with several concentrations up to the maximum non-toxic concentration of ES3 (1.56, 3.12, 6.25 and 12.5 μg/mL) or SM (3.12, 6.25, 12.5 and 25 μg/mL).

### LDH assay for cytotoxicity

To determine the maximum non-toxic concentration of *E. sativa* extracts and fractions, C2C12 myotubes, H4IIE hepatocytes and 3T3-L1 cells cultured in 12-well plates were treated with the extracts in concentrations ranging from 7.25 to 200 μg/mL or with the vehicle control (0.1% DMSO) for 18 h. At the end of the treatment, the cell culture media were removed and kept on ice for further analysis. The cells were then rinsed with phosphate buffer saline (PBS) and lysed with 1% Triton X-100, for 10 min. The activity of LDH in media (released LDH) and lysates (cellular LDH) was assessed using cytotoxicity detection LDH kit (Roche, Mannheim, Germany). The cytotoxicity was expressed as the ratio of released LDH to total LDH (total LDH = released LDH + cellular LDH) and was used to determine the maximum non-toxic concentration for each extract. Experiments were performed in triplicate.

### Glucose uptake assay

Stimulation of glucose uptake was measured in differentiated C2C12 cells. 18 h prior to the experiment, cells were incubated with *E. sativa* extracts and fractions. Insulin (100 nM) applied for 30 min and metformin (400 μM) applied for 18 h served as the positive controls, while 0.1% DMSO (the vehicle control, 18 h) was used as the negative control. After treatment, cells were rinsed twice with Krebs-phosphate buffer (KPB; 20 mM HEPES, 4.05 mM Na_2_HPO_4_, 0.95 mM NaH_2_PO_4_, pH 7.4, 136 mM NaCl, 4.7 mM KCl, 1 mM CaCl_2_, 1 mM MgSO_4_ and 5 mM glucose) and equilibrated in this buffer for 30 min at 37 °C. Following this, cells were washed twice with glucose-free KPB at 37 °C, then incubated with the same buffer containing 0.5 mCi/mL [^3^H] 2*-*deoxy*-*d*-*glucose (TRK-383, Amersham Biosciences, Buckinghamshire, UK). After 10 min, glucose uptake was stopped by the addition of cold KPB and cells were lysed with 0.1M NaOH. The cell lysates were transferred to scintillation vials for counting of ^3^H radioactivity. Non-specific uptake was measured in the presence of cytochalasin B (10 μM) and was subtracted from all values. Three replicates were performed for each experimental condition.

### Assessment of glucose 6-phosphatase (G6Pase) activity

After reaching 90% confluence, H4IIE hepatocytes were treated for 16 h in serum-free medium with either *E. sativa* extracts or fractions thereof. Some wells were treated with the vehicle (0.1% DMSO), which served as the negative control, or with 100 nM insulin, which served as the positive control. Cells were then washed in HEPES-buffered saline (10 mM HEPES, pH 7.4, 150 mM NaCl) at 37 °C. To assess G6Pase activity, the rate of glucose production in the presence of a non-limiting amount of glucose-6-phosphate (G6P) was determined. A commercial glucose assay kit (AutoKit Glucose; Wako Diagnostics, Richmond, VA) was used to measure total glucose production with modifications as described elsewhere (Heishi et al. 2006; Nachar et al. 2013). Briefly, AutoKit glucose buffer solution was diluted in water (1:4) and 200 μL was added to each well. To lyse the cells, 50 μL of 0.05% Triton X-100 in similarly diluted AutoKit Glucose buffer solution was used. G6P (20 μM final concentration) was then added to each well for a final volume of 275 μL. Consequently, the plates were incubated for 40 min at 37 °C and 500 μL of AutoKit Glucose colour reagent were added and the plates were further incubated for 5 min. Samples were then centrifuged at 3000 × *g* for 5 min at room temperature. The supernatant liquids were removed and the absorbance was measured at 505 nm at ambient temperature. Glucose concentrations were determined from a standard curve performed in parallel. For each treatment condition, control wells without exogenously applied G6P were included and absorbance measured from these wells was subtracted from absorbance measured in the presence of exogenous G6P. For all samples, protein content was measured using bicinchoninic acid assay (Simpson 2008) and G6Pase activity was expressed in relation to protein content. Data are expressed as mean ± SEM of three independent experiments, with four replicates per condition for each experiment.

### Stimulation of 3T3-L1 differentiation and measurement of intracellular triglyceride content

To test for *E. sativa* extracts’ glitazone-like activity, extracts were added to the differentiation media of the 3T3-L1 preadipocytes as described above. Vehicle (0.1% DMSO) in proliferation and differentiation media was used as negative controls and 10 μM rosiglitazone was used as the positive control. Eight days after induction of differentiation, intracellular triglycerdide content was quantified using AdipoRed reagent (Cambrex Bioscience Inc, Walkersville, MD) as previously described (Spoor et al. 2006).

### Preparation of the unsaponifiable fraction (USM), the saponifiable fraction (SM) and its ester derivatives fatty acid methyl esters (FAME)

Initial bioassays results described further below indicated that biological activity resided in the non-polar ES3 extract. It was thus chosen for further fractionation. To prepare the SM and the USM fractions, ES3 extract was saponified by refluxing with 10 mL alcoholic potassium hydroxide (10%). After distillation of alcohol and dilution with water, the USM was extracted several times with ether. The combined ethereal extracts were washed with distilled water until neutral to litmus paper and evaporated under reduced pressure using a rotavap. The residue left after evaporation of ether (USM fraction) was kept for further investigation. The aqueous mother liquor was acidified with 10% hydrochloric acid and the liberated fatty acids were extracted with ether several times. These ethereal extracts were combined, washed with distilled water until neutral to litmus paper and evaporated. The residue left after evaporation of ether constituted the saponifiable matter (SM). A part of SM was kept for screening of G6Pase and the remaining was subjected to methylation to afford FAME (Vogel 1967). Both SM and USM were subjected to biological screening for inhibition of G6Pase. Chemical investigation using GC/MS technique was also conducted to identify components of USM and FAME. A tentative identification of both USM and FAME components was performed based on the comparison of their relative retention time and mass spectra with those of the NIST, WILLY library data of the GC/MS system. The quantification of all identified components was based on peak area integration.

### GC/MS analysis of FAME and USM

One μL of FAME and USM were separately injected into a Thermo Scientific, Trace GC Ultra/ISQ Single Quadrupole MS, TG-5MS fused silica capillary column (30 m, 0.25 mm, 0.1 mm film thickness). For GC/MS detection, an electron ionization system with ionization energy of 70 eV was used. Helium was used as the carrier gas at a constant flow rate of 1 mL/min. The injector and MS transfer line temperature were set at 280 °C. For the analysis of USM, the oven temperature was set at an initial temperature of 50 °C for 2 min and then increased to 150 °C at a rate of 7 °C/min. The temperature was further raised to 270 °C at a rate of 5 °C/min, maintained for 2 min then further increased to 310 °C at a rate of 3.5 °C/min. For analysis of FAME, the oven temperature was maintained at 150 °C for 4 min then increased to 280 °C at a rate of 5 °C/min.

### Statistical analysis

All biological data are expressed as the mean ± SEM of three independent experiments with triplicate or quadruplicate analysis for each treatment. Results were analyzed by one-way analysis of variance (ANOVA). The EC_50_ and IC_50_ values were calculated by fitting the results to standard pharmacological dose-response algorithm using PRISM software version 6 (GraphPad Software Inc., La Jolla, CA). *p ≤* 0.05 is reported to be statistically significant.

## Results

### Percentage yield and cytotoxicity

**Figure 1.**
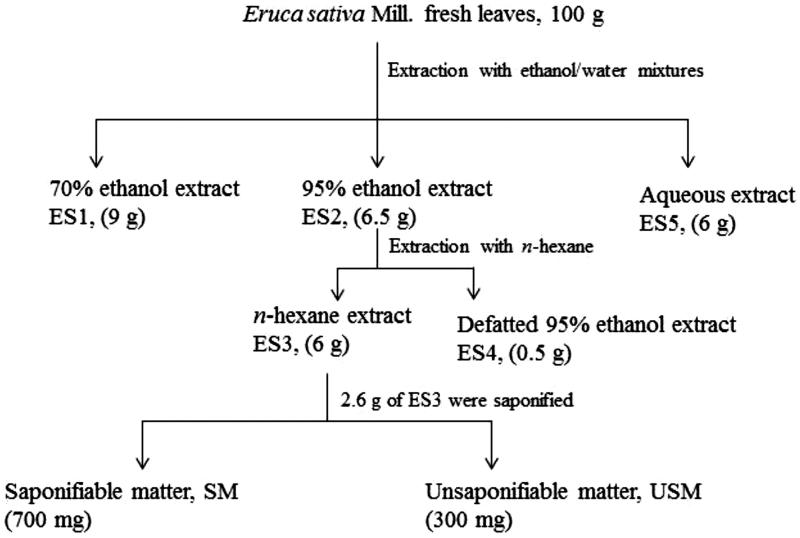
Preparation of *E. sativa* extracts and the phytochemical fractionation of the active *n*-hexane extract. Values in parentheses represent the yield of the different extracts and fractions.

ES1-ES5 extracts were prepared as described under materials and methods (Figure 1) and the % yield of the different extracts and fractions of *E. sativa* is presented in Table 1. The highest concentrations of extracts and fractions that induced less than 10% cytotoxicity were selected as the maximum non-toxic concentrations (Table 2).

**Table 2.** Maximum non-toxic concentrations of *E. sativa* Mill. extracts and fractions (μg/mL).

	Cell line		
Extract	H4-IIE	C2C12	3T3-L1
70% Ethanol (ES1)	25	50	50
95% Ethanol (ES2)	25	25	50
*n*-Hexane (ES3)	12.5	12.5	12.5
Defatted 95% ethanol (ES4)	50	50	100
Aqueous (ES5)	50	100	100
Saponifiable fraction (SM)	25	25	25
Unsaponifiable fraction (USM)	25	25	25

### ES3 extract and its fatty acid-rich SM fraction increase glucose uptake in C2C12 myotubes

**Figure 2.**
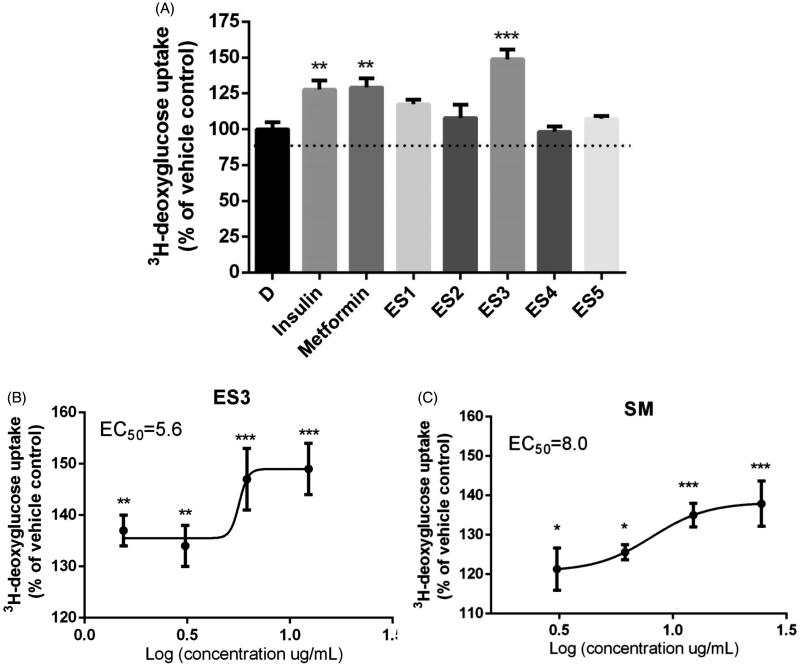
(A) Effect of *E. sativa* extracts on ^3^H-deoxyglucose uptake in C2C12 cells following 18 h treatment with their maximum non-toxic concentrations. Cells treated with insulin (100 nm, 30 min) or metformin (400 μM, 18 h) served as the positive control. (B) and (C) are dose-response analysis of the stimulation of glucose uptake by ES3 and SM, respectively. Cells were incubated with several concentrations of ES3 (1.56, 3.12, 6.25 and 12.5 μg/mL) or SM (3.12, 6.25, 12.5 and 25 μg/mL). Data are represented as mean ± SEM of three independent experiments and are expressed as % change in the rate of basal glucose uptakes relative to the vehicle control (0.1% DMSO). *p < 0.05, **p < 0.01, ***p < 0.001 significantly different from 0.1% DMSO.

After 18 h incubation with the plant extracts and fractions, only ES3 was able to enhance glucose uptake in C2C12 myotubes by 149 ± 5% at 12.5 μg/mL (*p* < 0.01), as compared to the vehicle control DMSO, (100%; Figure 2(A,B)). The ES3 fraction was then saponified to obtain SM and USM fractions. At 12.5 μg/mL, SM fraction stimulated glucose uptake by 135 ± 3% (*p* < 0.01, Figure 2(C)), relative to DMSO, whereas the USM fraction was inactive. The positive controls insulin and metformin stimulated glucose uptake by 128 ± 6% and 129 ± 7%, respectively (*p* < 0.01, Figure 2(A)), as compared to DMSO. EC_50_ values of ES3 and SM are 5.8 and 8.0 μg/mL, respectively.

### ES3 extract and SM fraction inhibit G6Pase activity in H4IIE hepatocytes following 16 h treatment

**Figure 3.**
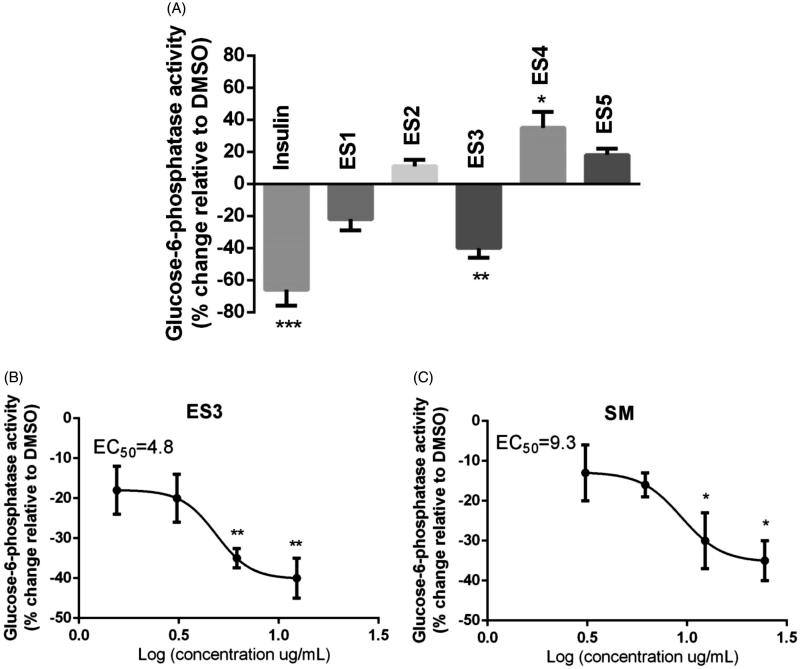
(A) Effect of *E. sativa* extracts on G6Pase activity in H4IIE hepatocytes. Cells were treated for 18 h with the maximum non-toxic concentrations of the indicated extracts. G6Pase activity was assessed colorimetrically by measuring glucose formation in the presence of a non-limiting amount of G6P as described under ‘Materials and Methods’. (B) and (C) are dose-response analysis of G6Pase inhibitory effects of ES3 and SM, respectively. Cells were separately treated with ES3 at different concentrations (1.56, 3.12, 6.25 and 12.5 μg/mL) or with SM (3.12, 6.25, 12.5 and 25 μg/mL). Data are expressed relative to vehicle control (0.1% DMSO, 0% inhibition). Cells treated with 100 nm insulin for similar time served as the positive control. Assays were carried out in triplicate.*p < 0.05, **p < 0.01 and ***p < 0.001 indicate a significant difference from vehicle control.

ES1-ES5 extracts were tested for the inhibition of G6Pase activity. ES3, applied at 12.5 μg/mL, significantly reduced the activity of G6Pase by 40 ± 5%, as compared to DMSO (set at 0%; *p* < 0.05, Figure 3(A,B)). Insulin (100 nM) applied for the same treatment period inhibited G6pase by 66 ± 10%, relative to DMSO. Interestingly, the SM fraction (12.5 μg/mL) was the only extract to suppress G6Pase by 30 ± 7%, relative DMSO (*p* < 0.05, Figure 3(C)). On the other hand, USM showed a strong tendency to inhibit G6Pase (by 27 ± 6% relative to DMSO; *p* = 0.06). IC_50_ values of ES3 and SM are 4.8 and 9.3 μg/mL, respectively.

### ES3 extract and SM fraction stimulate adipogenesis in 3T3-L1 adipocytes

**Figure 4.**
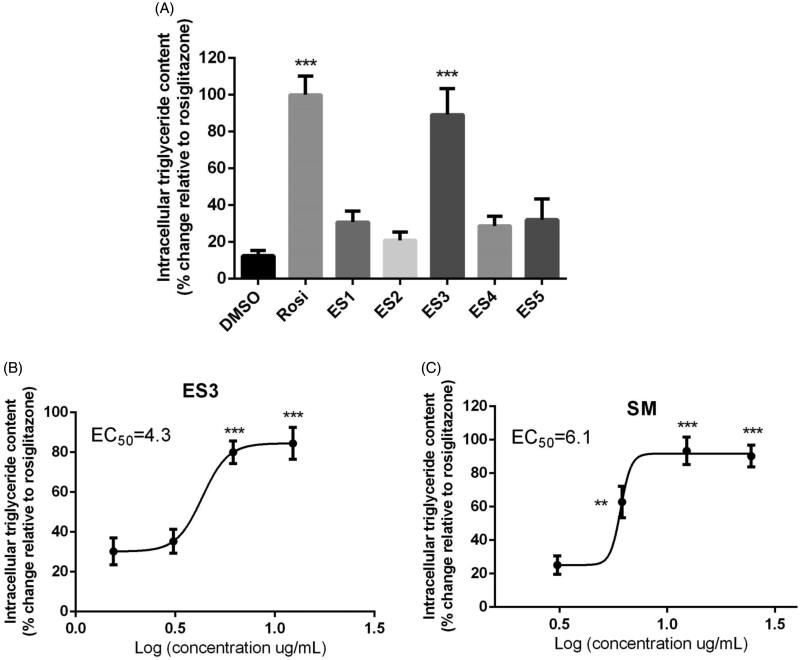
(A) Adipogenic activity of *E. sativa* extracts tested at their maximum non-toxic concentrations in 3T3-L1 adipocytes, as assessed by triglyceride content at the end of differentiation period. Two-day post-confluent preadipocytes were incubated with the plant extracts on day 0 of differentiation for 8 day. (B) and (C) are dose-response analysis of the adipogenic activities of ES3 and SM, respectively. Cells were incubated with several concentrations of ES3 (1.56, 3.12, 6.25 and 12.5 μg/mL) or SM (3.12, 6.25, 12.5 and 25 μg/mL). Data are represented as mean ± SEM and are expressed as % change in intracellular triglyceride content relative to the positive control rosiglitazone (10 μM), which was set at 100%. Assays were carried out in triplicate. **p < 0.01 and ***p < 0.001 indicate a significant difference from vehicle control. Rosi: rosiglitazone.

Accumulation of intracellular triglycerides in differentiating adipocytes has been considered as a marker of adipogenesis. At day 8 of differentiation, only ES3 extract and its SM fraction showed a significant increase in intracellular triglyceride content in 3T3-L1 adipocytes as compared to DMSO (*p* < 0.001, Figure 4(A–C)). The adipogenic activities were similar to that of rosiglitazone (set at 100%, Figure 4(A–C)). EC_50_ values for ES3 and SM are 4.3 and 6.1 μg/mL, respectively.

### Identification of SM and USM components using GC/MS technique

GC/MS analysis of SM resulted in the identification of 24 compounds (Table 3) representing 94.78% of the FAME prepared from the SM fraction. The content of identified saturated fatty acids was higher than that of the identified non-saturated ones (63.3% and 31.2% of FAME, respectively). The major identified saturated fatty acids were palmitic acid and azelaic acid (43.3% and 15.9%, respectively), while the main identified unsaturated fatty acids were *trans*-vaccenic acid (14.3%) and palmitoleic acid (4.2%).

**Table 3.** GC/MS analysis of the SM of the *n*-hexane extract of *E. sativa* Mill. leaves.

Rt	Compound	Molecular weight	Molecular formula	Base beak	Peak area %
6.32	Azelaic acid	216	C_11_H_20_O_4_	74	15.99
8.03	Sebacic acid	230	C_12_H_22_O_4_	74	0.56
8.39	Linolenic acid (18:3)	292	C_19_H_32_O_2_	85	0.42
9.45	Pentadecylic acid (15:0)	270	C_17_H_34_O_2_	74	0.52
10.37	Undecanedioic acid	244	C_13_H_24_O_4_	74	0.4
13.02	11-Hexadecenoic acid	268	C_17_H_32_O_2_	74	1.28
13.65	Palmitic acid	270	C_17_H_34_O_2_	74	43.29
14.17	Dimethyl bicycle (3.3.0) octan-3,7-dione-4,6-dicarboxylate	254	C_12_H_14_O_6_	71	0.32
14.57	Palmitoleic acid (16:1)	268	C_17_H_32_O_2_	87	4.22
14.73	1-Eicosanol	298	C_20_H_42_O	57	1.05
14.88	Hexadecanoic acid	284	C_18_H_36_O_2_	57	0.29
15.11	10-Hydroxy-2-methyl-3-oxodecanoic acid	230	C_12_H_22_O_4_	55	0.77
15.3	1,3-Dioxolane, 2,2-dimethyl-4-(2-caromethoxy-5-methylene-1-cyclopentyl)	240	C_13_H_20_O_4_	101	1.09
15.4	Hexadecanoic acid	284	C_18_H_36_O_2_	74	1.04
15.76	1,2,3,4-Tetrahydro-1,1,4,4,5-pentamethyl-6-phenylanthracene	328	C_25_H_28_	145	1.15
16.9	*trans*-Vaccenic acid (18:1)	296	C_19_H_36_O_2_	74	14.29
17.27	(1,4,7)-3,3-Dimethoxy-7-methoxycarbonyl-7-methyl-5-(2,5-dioxacyclopentyl) bicycle(2,2,2)oct-5-en-2-one	326	C_16_H_22_O_7_	75	6.12
17.52	7,10-Octadecadienoic	294	C_19_H_34_O_2_	95	0.19
17.67	5,16-Octadecadien-1-ol acetate	308	C_17_H_24_O_5_	95	0.24
20.58	Arachidic acid (20:0)	326	C_21_H_42_O_2_	87	0.24
22.46	*cis*-Vaccenic acid (18:1)	282	C_18_H_34_O_2_	57	0.21
23.73	Behenic acid (22:0)	354	C_23_H_46_O_2_	87	0.37
26.66	Lignoceric acid (24:0)	382	C_25_H_50_O_2_	74	0.54
28.53	Tricosanoic acid (23:0)	398	C_25_H_50_O_3_	57	0.19
Total identified fatty acid methyl ester					94.78
Total identified saturated fatty acid methyl ester					63.33
Total identified unsaturated fatty acid methyl ester					31.2

On the other hand, 29 compounds were identified in USM representing (94.7%) of total USM (Table 4). The identified sterols and non-sterol components were found to represent 5.2% and 89.4%, respectively. β-Sitosterol was the major identified sterol (3.2%) followed by stigmasterol (0.9%). 6,10,14-Trimethyl-2-pentadecanone (30.3%) was the major identified non-sterol compound followed by 3,7,11,15-tetramethyl-2-hexadecen-1-ol (19.1%).

**Table 4.** GC/MS analysis of the USM of the *n*-hexane extract of *E. sativa* Mill. leaves.

Rt	Compound	Molecular weight	Molecular formula	Base beak	Peak area %
26.16	1-Hexadecanol	242	C_16_H_34_O	83	0.99
27.3	2-Hexyldodecyl acetate	312	C_20_H_40_O_2_	97	0.45
28.02	*E*-8-Tridecen-2-ol, acetate	240	C_15_H_28_O_2_	57	0.91
29.23	1-Nonadecanol	284	C_19_H_40_O	69	0.31
30.6	1-Octadecanol	270	C_18_H_38_O	97	3.24
31.88	6,10,14-Trimethyl-2-pentadecanone,	268	C_18_H_36_O	109	30.32
32.09	Octadecanal	268	C_18_H_36_O	96	0.49
32.59	2,5-Furandione, dihydro-3-dodecenyl	266	C_16_H_26_O_3_	69	0.82
33.13	2(3H)-Furanone, dihydro-5-tetradecyl-	282	C_18_H_34_O_2_	57	4.01
33.24	8-Hexadecenal, 14-methyl	252	C_17_H_32_O	57	1.8
33.72	Isophytol	296	C_20_H_40_O	71	0.37^a^
34.23	1,8-Dipropoxyanthraquinone	324	C_20_H_40_O_4_	99	0.89
34.62	Behenic alcohol	326	C_22_H_46_O	83	3.23
35.37	11-Methyl-12-tetradecen-1-ol acetate	268	C_17_H_32_O_2_	83	0.94
36.33	2-Acetoxy-1,1,10-trimethyl-6,9-epidioxydecalin	268	C_15_H_24_O_4_	85	1.16
36.67	Phytol	296	C_20_H_40_O	71	2.27^a^
38.25	1-Tricosanol	340	C_23_H_48_O	83	1.72
38.71	3,5-Cyclo-5-cholestan-6-ol	387	C_26_H_45_NO	157	0.83
39.53	Methyl 2-octylcyclopropene-1-heptanoate	294	C_19_H_34_O_2_	71	8.1
41.6	1-Hexadecanol	382	C_26_H_54_O	97	1.86
43.26	Pentacosane	352	C_25_H_52_	71	0.4
44.25	3,7,11,15-Tetramethyl-2-hexadecen-1-ol	296	C_20_H_40_O	149	19.09
44.72	1-Pentacosanol	368	C_25_H_52_O	69	0.48
46.25	2-Tetracosanol, acetate	396	C_206_H_52_O_2_	71	4
46.94	1-Hexacosanol	382	C_26_H_54_O	97	0.77
52.11	Cholesterol	386	C_27_H_46_O	105	0.33
53.52	5,10-Secocholest-1(10)-en-3,5-dione	400	C_27_H_44_O_2_	105	0.77
53.83	β-Sitosterol	414	C_29_H_50_O	95	3.19
53.95	Stigmasterol	412	C_29_H_48_O	81	0.95
Total identified compounds					94.69
Total identified sterols					5.24
Total identified non-sterol components					89.45

a Compounds could be artefacts from the solvents.

## Discussion

*Eruca sativa* leaf or rocket salad is a popular Mediterranean salad vegetable that has demonstrated some antidiabetic potential. Indeed, a polyherbal preparation including *E. sativa* leaves reduced fasting blood glucose and hepatic steatosis in streptozotocin-induced diabetic rats when administered by oral gavage (Sajeeth et al. 2010). Similarly, daily oral administration of *E. sativa* seed oil for 2 weeks decreased hyperglycaemia, improved lipid profile and stimulated glutathione production in the liver of alloxan-induced diabetic rats (El-Missiry & El Gindy 2000). In addition, ethanol and aqueous extracts of *E. sativa* reduced the activity of carbohydrate-hydrolyzing enzymes namely α-amylase, α-glucosidase and β-galactosidase (Hetta et al. 2014). As T2DM is a complex disorder, we aimed to investigate further this potential by examining the effect of extracts prepared from *E. sativa* leaves on cell lines derived from the major insulin-responsive tissues, namely skeletal muscle, liver and adipose tissue.

Skeletal muscle is the major site of glucose disposal, accounting for about 80% of total insulin-stimulated glucose uptake. Impaired insulin sensitivity in skeletal muscle leads to decreased postprandial glucose uptake. This precedes the onset of T2DM by several years and is considered the major defect in the disease (Abdul-Ghani & DeFronzo 2010). In this study, we used organic solvents of varying polarity to determine the potential antidiabetic activity of polar and non-polar components of *E. sativa*. We first examined glucose uptake in C2C12 myotubes in culture and evaluated the potential stimulatory effect of five extracts of *E. sativa* leaves (70% ethanol, 95% ethanol, *n*-hexane, defatted 95% ethanol and aqueous extracts). Interestingly, the hexane-soluble fraction of *E. sativa* extract (ES3) and its fatty acid-rich fraction, which represent 26.9% of ES3, significantly increased basal glucose uptake in C2C12 myotubes to levels similar or higher than those of the positive controls insulin and metformin, with EC_50_ values of 5.8 and 8.0 μg/mL, respectively.

Liver is another major regulator of glucose homeostasis and whole-body energy balance. As brain is incapable of synthesizing or storing glucose, maintenance of blood glucose levels is primordial for normal brain functions. During prolonged fasting, *de novo* glucose synthesis (gluconeogenesis) is mainly carried out by the liver from gluconeogenic substrates such as lactate, pyruvate and amino acids (Rui 2014). Gluconeogenesis involves several steps; the last and rate-limiting step is catalyzed by the enzyme G6Pase and involves the dephosphorylation of G6P to generate glucose (Rui 2014). In the fed-state, insulin exerts an inhibitory effect on hepatic glucose production mainly through downregulation of the key gluconeogenic gene *G6Pase*. In T2DM, insulin’s inhibitory effect on hepatic gluconeogenic enzymes is attenuated, which results in increased hepatic glucose output and fasting hyperglycaemia (Wilcox 2005). Therefore, targeting the key gluconeogenic enzyme is a promising avenue for the treatment of T2DM. To evaluate the effect of plant extracts on hepatic glucose output, their effect on G6Pase was assessed in cultures of H4IIE liver cells. ES3 and SM were the only fractions that exhibited an important inhibitory activity on G6Pase with IC_50_ values of 4.8 and 9.3 μg/mL, respectively.

Finally, the glitazone-like activity of the plant extracts was assessed in cultured 3T3-L1 adipocytes. Thiazolidinediones (or glitazones) are a class of oral antidiabetic compounds that exert their antidiabetic activity mainly through the stimulation of adipogenesis. This promotes the uptake of free fatty acid into adipocytes, thus reducing ectopic fat deposition in skeletal muscle and liver, which is manifested as enhanced insulin sensitivity and reduced hyperglycaemia (Chiarelli & Di Marzio 2008). Similar to their effect on skeletal muscle and hepatic cell lines, ES3 and SM were able to induce adipogenesis to levels comparable to that of the positive thiozolidinedione control, rosiglitazone. EC_50_ values are equal to 4.3 and 6.1 μg/mL, respectively.

Care was also taken to identify the kind of fatty acids present in our non-polar extracts since saturated fatty acids have generally been suspected to be detrimental to health and to worsen diabetic situations (Boden & Shulman 2002; Boden 2003), albeit this is a matter of continuing debate (discussed further below). The major components of the SM fraction of ES3 were found to be two saturated fatty acids, namely palmitic acid and azelaic acid and two unsaturated fatty acids, namely trans-vaccenic and palmitoleic acids. Several studies have reported beneficial effects of azelaic (saturated), *trans*-vaccenic (unsaturated) and palmitoleic (unsaturated) acids for metabolic disorders, notably through the improvement of insulin sensitivity and the reduction of hyperlipidaemia (Wang et al. 2008; Duckett et al. 2014; Hirahatake et al. 2014; Muthulakshmi et al. 2015). On the other hand, administration of palmitic acid (saturated) to laboratory animals was found to induce a state of insulin resistance. However, the risk-benefit of human consumption of vegetable oil rich in palmitic acid such as palm oil is the subject of continuing scientific debate. Several studies have shown that moderate consumption of palm oil does not increase serum cholesterol levels (Odia et al. 2015). Other studies even reported the anti-atherogenic and protective effect of palm oil against heart and blood vessel ischaemic injuries (Odia et al. 2015).

Based on the results of the present study, it can be concluded that the beneficial effect of the hexane fraction of *E. sativa* extract and its fatty acid-rich fraction on muscle glucose uptake, hepatic G6Pase activity and adipocyte differentiation in cultured murine cells is principally associated with the combination of fatty acids contained in the SM fraction of the hexane extract. However, further studies will be necessary to determine the specific contribution of these various fatty acid components to the observed biological activities of the plant. Nevertheless, our studies support the use of *E. sativa* leaves as a potential nutraceutical product for the prevention and treatment of T2DM and provide impetus for future clinical studies.
